# Trajectories of chronic disease burden, depressive symptoms, and functional limitations related to falls in middle-aged and older adults: a matched longitudinal study

**DOI:** 10.3389/fnagi.2026.1896362

**Published:** 2026-08-18

**Authors:** Mengmeng Wang, Yaohui Cui, Lincheng Duan, Xiaowei Qin

**Affiliations:** 1 Heping Hospital Affiliated to Changzhi Medical College, Changzhi, China; 2 Chengdu University of Traditional Chinese Medicine, Chengdu, China; 3 Heji Hospital Affiliated to Changzhi Medical College, Changzhi, China

**Keywords:** activities of daily living, aging, chronic disease burden, depressive symptoms, falls, longitudinal trajectories

## Abstract

**Background:**

Falls are often treated as isolated events, but the physiological, psychological, and functional trajectories surrounding a first fall remain poorly understood. We examined chronic disease burden, depressive symptoms, and activities of daily living (ADL) limitations before and after first fall reports in England and the United States.

**Methods:**

We conducted a matched longitudinal analysis of adults aged ≥50 years in the English Longitudinal Study of Ageing (ELSA) and the Health and Retirement Study (HRS). Participants who first reported a fall during follow-up were matched 1:1 to participants without reported falls using coarsened exact matching (CEM). Matching used baseline age, birth year, sex, and education. Repeated outcomes comprised an eight-condition count, the eight-item Center for Epidemiologic Studies Depression (CES-D) scale, and a six-item ADL count. Time zero was the first fall-report wave or matched pseudo-index wave. Outcome-specific piecewise mixed-effects models incorporated quadratic time terms selected using likelihood-ratio tests and information criteria.

**Results:**

Matched samples included 3,582 ELSA and 4,178 HRS participants. At time zero, adjusted fall-group minus non-fall-group differences were 0.10, 0.30, and 0.12 in ELSA. The corresponding differences in HRS were 0.20, 0.35, and 0.28. These values represent chronic condition, depressive symptom, and ADL limitation counts, respectively. At +8 years, the corresponding differences were 0.19, 0.33, and 0.30 in ELSA. In HRS, they were 0.40, 0.43, and 0.95. Nonlinear specifications improved fit for all outcomes in both cohorts. Dichotomous analyses also showed higher odds of multimorbidity, depression, and ADL disability in the fall group.

**Conclusion:**

A first fall report marked a period of accumulating physiological, psychological, and functional vulnerability in both cohorts. Because time zero was a survey report wave rather than the exact fall date, these event-aligned associations do not isolate acute causal effects. Nevertheless, a first fall report may provide a useful trigger for comprehensive assessment and sustained functional support.

## 1 Introduction

Falls are an increasingly important concern in aging societies. They are common among older adults worldwide (Alawlah et al., 2026; Wang et al., 2026). Their consequences extend beyond the immediate injury and include fractures, hospitalization, disability, long-term care use, death, and lasting losses in autonomy and quality of life (Cunningham et al., 2020; Vaishya and Vaish, 2020; Lohman et al., 2018). The World Health Organization estimates that 37.3 million falls require medical care annually, with the greatest fatal burden among adults aged 60 years or older (World Health Organization, 2021). In the United States, more than 14 million adults aged 65 years or older report a fall each year, representing about one quarter of this age group (Centers for Disease Control and Prevention, 2026). Despite growing prevention efforts, fall-related mortality has risen by 30% over the past decade (Castle, 2019). Preventing falls and improving post-fall care are therefore essential to preserving independence and wellbeing in later life (Montero-Odasso et al., 2022; Ganz and Latham, 2020).

Falls should not, however, be viewed solely as isolated accidental injuries. They often occur alongside accumulating chronic conditions, poor mental health, and declining functional independence (Ganz and Latham, 2020; Stel et al., 2003; Ambrose et al., 2013). Falls may also coincide with, or contribute to, further deterioration across these domains. A fall can therefore serve as an observable clinical marker within a broader process of health vulnerability, rather than simply reflecting external hazards or chance. Chronic disease burden, depressive symptoms, and ADL limitations capture physiological, psychological, and functional health, respectively. Examining them together may clarify the broader clinical significance of falls.

Existing evidence links multimorbidity, depressive symptoms, and ADL disability to both fall risk and adverse post-fall outcomes. Chronic conditions may increase fall risk through impaired balance, reduced muscle strength, sensory deficits, and slower reactions (Ambrose et al., 2013; Schwartz et al., 2008; Fan et al., 2025). Depressive symptoms and fear of falling may promote activity avoidance, reduced social participation, and functional decline. These processes can reinforce a cycle of falls, fear, activity restriction, functional deterioration, and recurrent falls (Delbaere et al., 2010; Chen et al., 2023; Lim et al., 2025; Izquierdo et al., 2025). ADL disability is an important consequence of falls, but it may also develop beforehand. Reduced mobility, self-care capacity, and adaptation to environmental challenges may thereby increase fall risk (Zhang et al., 2020; Pereira et al., 2020; Himes and Reynolds, 2011).

Important gaps nevertheless remain. Most studies have assessed falls or related outcomes at a single time point, obscuring whether adverse changes precede falls, follow them, or both. Chronic disease burden, depressive symptoms, and ADL disability have also usually been examined separately rather than within one longitudinal framework. Event-centered approaches have characterized trajectories around pain onset, smoking cessation, continuing education, cardiovascular events, and dementia diagnosis (Bloomberg et al., 2025; Bloomberg et al., 2025; Bloomberg et al., 2026; Vishwanath et al., 2026; Wu et al., 2025). Here, a trajectory denotes the model-estimated pattern of repeated outcomes over time aligned to an index wave, not membership in a latent class. Modeling outcomes from 8 years before to 8 years after the index wave allows pre-index accumulation and post-index change to be evaluated within the same framework.

We compared England and the United States because ELSA and HRS provide closely harmonized, nationally representative aging data from distinct health-system contexts. England’s tax-funded National Health Service provides universal coverage based primarily on need. The United States uses a mixed public-private system with more heterogeneous coverage and financial access (Zaninotto et al., 2020; Anderson et al., 2022; Rice et al., 2020). Replication across these settings tests the robustness of the overall trajectory pattern, but does not identify a causal effect of health-system design.

Using a matched longitudinal design, we examined whether the three health indicators differed before the first fall-report wave and whether those differences subsequently widened. We also assessed whether the patterns were reproduced across cohorts. The analysis positioned first fall reports within a continuum of multidimensional vulnerability and explored opportunities for early assessment and sustained support.

## 2 Methods

### 2.1 Study design and population

We conducted a matched longitudinal analysis using prospective data from ELSA and HRS (Steptoe et al., 2012; Sonnega et al., 2014). Both are large, nationally representative cohorts of middle-aged and older adults, with interviews approximately every 2 years. They repeatedly assess demographic and socioeconomic characteristics, health behaviors, chronic conditions, mental health, falls, and functional status. These features support the examination of health trajectories across aging populations.

**Figure 1:**
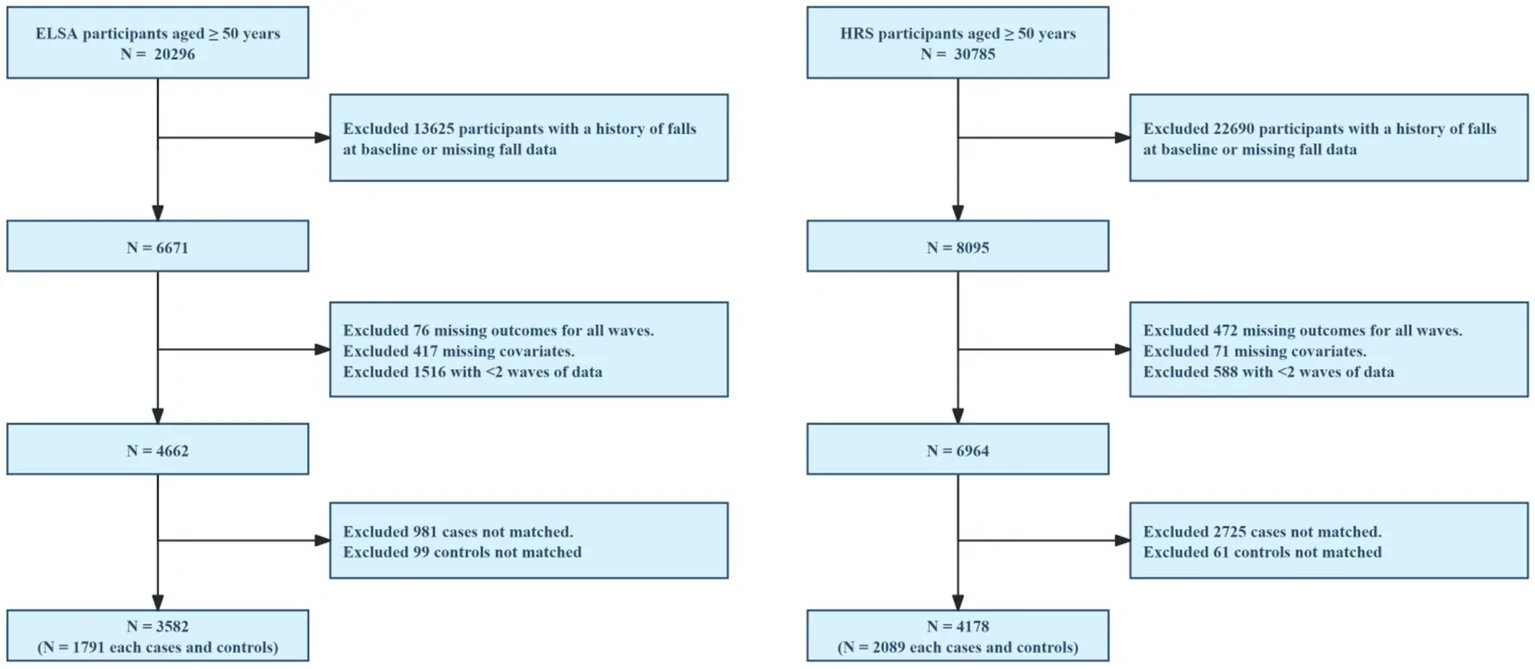
Participant selection flowchart for the ELSA and HRS matched cohorts.

We used waves 1–10 of ELSA and waves 7–15 of HRS. Analytical baseline was each participant’s first wave with available fall status, main outcomes, and key covariates. We excluded participants who reported a fall or lacked fall information at baseline. Eligible participants were aged 50 years or older, had no baseline fall report, and had repeated measurements for at least one main outcome. We further excluded those missing all outcome data, missing key covariates, or contributing fewer than two follow-up waves (Figure 1).

The ELSA and HRS received approval from the relevant ethics committees, and all participants provided written informed consent. This secondary analysis used anonymized, publicly available data and therefore required no additional ethical approval.

### 2.2 Fall assessment and study groups

At each wave, both ELSA and HRS asked participants whether they had fallen since the previous interview. Responses were harmonized into a binary indicator of any reported fall. Among participants with no baseline fall report, those reporting a fall for the first time during follow-up formed the fall group. The wave of the first fall report defined time zero. Because the question covered the interval since the previous interview, the fall could have occurred at any point during the approximately two-year interval. Time zero therefore represents a report wave, not the exact event date.

### 2.3 Outcomes

The main outcomes were chronic disease burden, depressive symptoms, and ADL limitations. These represented physiological, psychological, and functional health, respectively, and were repeatedly assessed across survey waves.

Chronic disease burden was quantified as the number of self-reported physician-diagnosed conditions recorded in both cohorts (Abbad-Gomez et al., 2025). The harmonized list comprised hypertension, diabetes, cancer, chronic lung disease, heart disease, stroke, psychiatric conditions, and arthritis. This count captured the accumulation of chronic conditions over time. We also defined multimorbidity as two or more chronic conditions.

Depressive symptoms were assessed using the shortened Center for Epidemiologic Studies Depression (CES-D) scale (Turvey et al., 1999); higher scores indicated more depressive symptoms. We also derived a binary depression outcome using the established CES-D cut-off commonly applied in ELSA and HRS analyses.

Functional status was assessed using six basic ADL items (Landré et al., 2025). Participants were asked about difficulties with dressing, bathing, eating, transferring in or out of bed, toileting, and walking across a room. Each reported difficulty or need for help was counted as one limitation, and the total score represented the number of impaired ADL tasks. A binary ADL disability variable was additionally created, indicating whether the participant had difficulty with at least one ADL item.

### 2.4 Covariates

We selected baseline covariates that could influence both subsequent fall reports and outcome trajectories. They covered demographic characteristics, socioeconomic position, existing health conditions, and health behaviors. Age, birth year, sex, education, and household wealth characterized demographic and socioeconomic position. Education was harmonized across cohorts as low, intermediate, or high, and wealth was divided into cohort-specific tertiles.

Baseline health status comprised self-reported physician-diagnosed hypertension, diabetes, cancer, chronic lung disease, heart disease, stroke, psychiatric conditions, and arthritis. Health behaviors included smoking, alcohol consumption, and moderate-to-vigorous physical activity at least weekly. All covariates were measured at analytical baseline. Because chronic condition count was itself an outcome, its models did not additionally adjust for the component diseases. Models of depressive symptoms and ADL limitations adjusted for baseline health conditions and behaviors to reduce residual group differences.

### 2.5 Matching

To reduce baseline differences between participants who later reported falls and those who did not, we created matched samples separately in ELSA and HRS using CEM (Iacus et al., 2012). Eligible controls remained free of reported falls through the risk period corresponding to their matched case’s first fall-report wave. CEM formed strata from coarsened baseline covariates and retained fall and non-fall participants represented within the same strata.

Matching used baseline age, birth year, sex, and education (Bloomberg et al., 2025). Age and birth year were coarsened into five-year categories; sex and harmonized education level were matched exactly. The k-to-k option in the Stata cem package sampled equal numbers of cases and controls within each stratum. This procedure yielded a 1:1 design without matching weights (Blackwell et al., 2009). We assessed covariate balance before and after matching using standardized mean differences (SMDs) and variance ratios. An absolute SMD < 0.10 and a variance ratio near 1 indicated acceptable balance. Diagnostics for all baseline variables appear in Supplementary Table A1.

### 2.6 Statistical analysis

We summarized baseline characteristics in each matched cohort using means with standard deviations or counts with percentages. Group comparability was evaluated using SMDs rather than significance tests, which depend strongly on sample size. We then examined observed and model-adjusted trajectories of chronic disease burden, depressive symptoms, and ADL limitations around the index wave.

For the main analyses, a trajectory was the model-estimated mean outcome from 8 years before to 8 years after the index wave. Follow-up time was aligned to the first fall-report wave for the fall group and the matched pseudo-index wave for controls. Negative and positive time segments represented pre-index and post-index periods, respectively. Mixed-effects models included fall status, both time segments, their interactions with fall status, and baseline covariates. Models also included participant-specific random intercepts and random slopes for both segments, with an unstructured covariance matrix.

We compared the initial piecewise linear model with nested, outcome-specific quadratic models using likelihood-ratio tests fitted by maximum likelihood, Akaike information criterion (AIC), Bayesian information criterion (BIC), and parsimony. Quadratic terms were retained when they materially improved fit, including group-specific terms when supported. Supplementary Table A2 reports the selected forms and fit statistics. Because slope contrasts vary over time in quadratic models, we focused on adjusted fall-group minus non-fall-group differences at −8, 0, and +8 years, with 95% confidence intervals.

**Figure 2:**
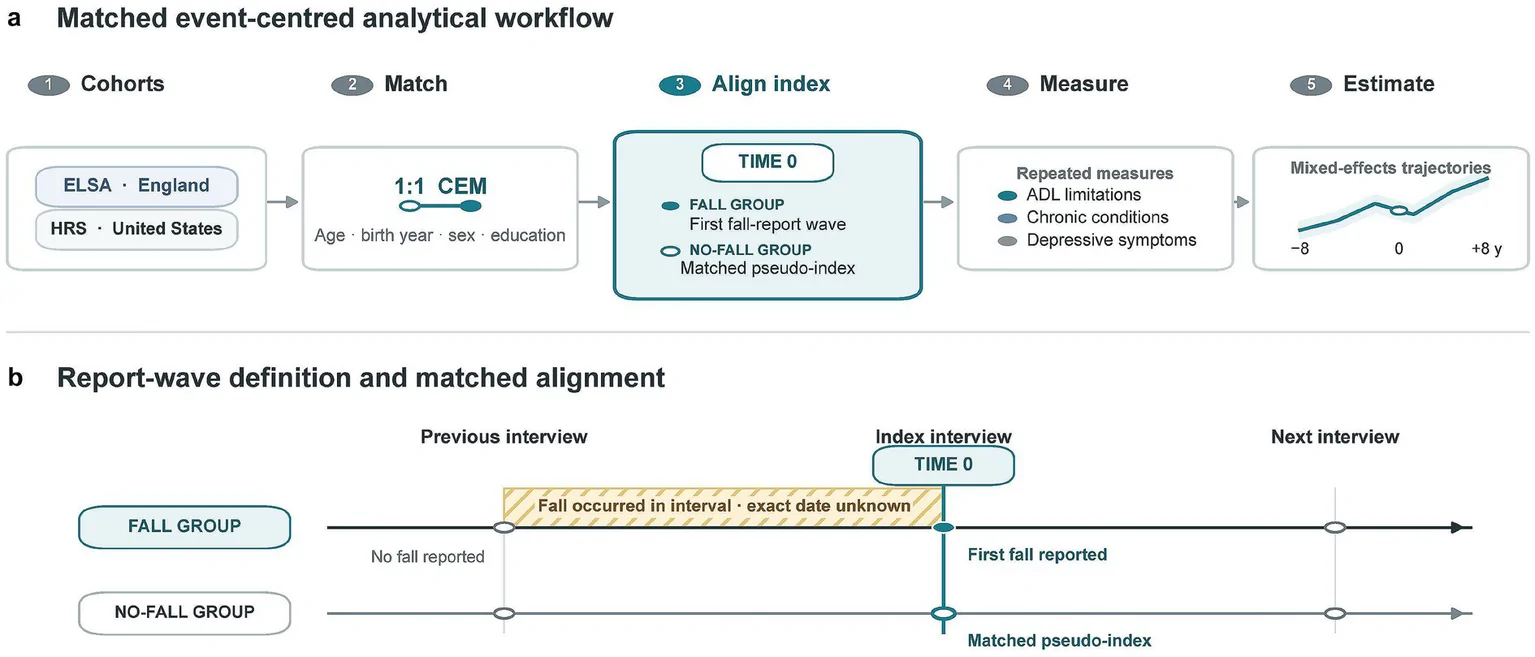
Matched event-centered analytical workflow and definition of time zero. **(a)** Participants in ELSA and HRS were matched using CEM and aligned at the first fall-report or matched pseudo-index wave. Repeated measures captured chronic disease burden, depressive symptoms, and ADL limitations. **(b)** A fall could have occurred at any time since the previous interview. Time zero therefore represents the report wave, not the exact fall date. CEM, coarsened exact matching; CES-D, Center for Epidemiologic Studies Depression scale; ADL, activities of daily living.

For clinical interpretation, we also fitted piecewise logistic mixed-effects models for multimorbidity, depression, and ADL disability. These outcomes were defined as at least two chronic conditions, a CES-D score ≥3, and at least one ADL limitation, respectively. The models used the same covariate structure as the continuous-outcome analyses and included a participant-level random intercept. Logistic mixed-effects odds ratios (ORs) are conditional on individual random effects, so model-based predictions were used to estimate population-average ORs. Details of the conversion are provided in Supplementary Methods A1. The non-fall group was the reference. We reported ORs with 95% confidence intervals (CIs) at −8, 0, and +8 years. The overall matched event-centered workflow and the wave-based definition of time zero are summarized in Figure 2.

### 2.7 Subgroup analyses

We examined whether fall-related trajectory patterns varied by age, sex, education, and household wealth. Age was classified as <75 or ≥75 years at the first fall-report wave or matched pseudo-index wave. Education and wealth used the harmonized categories described above. For each factor, we added interactions with fall status and time and fitted models separately in ELSA and HRS.

### 2.8 Sensitivity analyses

Robustness was assessed by restricting analyses to participants with at least three, four, or five survey waves. These restrictions examined whether selective attrition or survival influenced the estimated trajectories.

Statistical analyses were conducted in Stata 18. All tests were two-sided, and *p* < 0.05 was considered statistically significant.

## 3 Results

### 3.1 Participant characteristics

In ELSA, 20,296 adults aged 50 years or older were initially identified. Excluding 13,625 with baseline falls or missing fall data left 6,671 participants. We then excluded 76 with no outcome data across waves, 417 with missing covariates, and 1,516 with fewer than two follow-up waves. Of the 4,662 participants entering matching, 981 unmatched fall cases and 99 unmatched controls were excluded. The final matched sample comprised 3,582 participants, with 1,791 in each group.

In HRS, 30,785 adults aged 50 years or older were initially identified. Excluding 22,690 with baseline falls or missing fall data left 8,095 participants. We then excluded 472 with no outcome data across waves, 71 with missing covariates, and 588 with fewer than two follow-up waves. Of the 6,964 participants entering matching, 2,725 unmatched fall cases and 61 unmatched controls were excluded. The final matched sample comprised 4,178 participants, with 2,089 in each group.

CEM achieved near-exact balance on the four matching variables. The maximum absolute SMD across their levels was 0.004 in ELSA and 0.006 in HRS. All measured baseline characteristics in ELSA had absolute SMDs <0.10. In HRS, residual differences remained for arthritis (SMD = 0.142), chronic condition count (SMD = 0.111), and multimorbidity (SMD = 0.110). The SMD for psychiatric conditions was 0.085. We therefore retained these variables as covariates where appropriate and did not describe the HRS sample as fully balanced (Tables 1, 2 and Supplementary Tables A1, A3).

**Table 1:** Baseline sociodemographic and behavioral characteristics of the matched ELSA and HRS cohorts.

Characteristic	ELSA (*n* = 3,582)			HRS (*n* = 4,178)		
	Non-fall	Fall	|SMD|	Non-fall	Fall	|SMD|
Age, mean (SD), years	69.2 (6.6)	69.2 (6.6)	0.004	73.0 (6.7)	72.9 (6.8)	0.006
Birth year, mean (SD)	1933.6 (7.6)	1933.6 (7.6)	0.002	1931.0 (7.0)	1931.0 (7.1)	0.003
Female, *n* (%)	801 (44.7)	801 (44.7)	0.000	1,046 (50.1)	1,046 (50.1)	0.000
Low education, *n* (%)	968 (54.0)	968 (54.0)	0.000	582 (27.9)	582 (27.9)	0.000
Intermediate education, *n* (%)	659 (36.8)	659 (36.8)	0.000	1,145 (54.8)	1,145 (54.8)	0.000
High education, *n* (%)	164 (9.2)	164 (9.2)	0.000	362 (17.3)	362 (17.3)	0.000
Low wealth, *n* (%)	543 (30.3)	467 (26.1)	0.094	617 (29.5)	593 (28.4)	0.025
Intermediate wealth, *n* (%)	626 (35.0)	617 (34.5)	0.011	660 (31.6)	722 (34.6)	0.063
High wealth, *n* (%)	622 (34.7)	707 (39.5)	0.098	812 (38.9)	774 (37.1)	0.037
Weekly MVPA, *n* (%)	1,364 (76.2)	1,389 (77.6)	0.033	1,530 (73.2)	1,556 (74.5)	0.028
Alcohol consumption, *n* (%)	1,586 (88.6)	1,583 (88.4)	0.005	969 (46.4)	946 (45.3)	0.022
Current smoking, *n* (%)	278 (15.5)	234 (13.1)	0.070	253 (12.1)	209 (10.0)	0.067

Values are mean (SD) or *n* (%). |SMD| denotes the absolute standardized mean difference.

**Table 2:** Baseline health and functional characteristics of the matched ELSA and HRS cohorts.

Characteristic	ELSA (*n* = 3,582)			HRS (*n* = 4,178)		
	Non-fall	Fall	|SMD|	Non-fall	Fall	|SMD|
Chronic condition count, mean (SD)	1.1 (1.0)	1.1 (1.1)	0.018	1.8 (1.3)	2.0 (1.3)	0.111
Multimorbidity, *n* (%)	557 (31.1)	566 (31.6)	0.011	1,180 (56.5)	1,293 (61.9)	0.110
CES-D score, mean (SD)	1.2 (1.8)	1.3 (1.7)	0.046	1.1 (1.6)	1.3 (1.8)	0.097
Depression, *n* (%)	315 (17.7)	345 (19.4)	0.044	300 (15.0)	366 (18.4)	0.091
ADL limitation count, mean (SD)	0.3 (0.8)	0.3 (0.8)	0.058	0.2 (0.8)	0.2 (0.7)	0.021
ADL disability, *n* (%)	262 (14.6)	318 (17.8)	0.085	208 (10.0)	254 (12.2)	0.070

Values are mean (SD) or *n* (%). |SMD| denotes the absolute standardized mean difference. Individual diagnoses are reported in Supplementary Table A3.

### 3.2 Trajectories of chronic disease burden

Outcome-specific quadratic models fitted the continuous trajectory data better than the corresponding piecewise linear models in both cohorts. Across comparisons, likelihood-ratio tests yielded *p* = 0.002 or *p* < 0.001 (Supplementary Table A2). After adjustment, chronic condition counts increased in both groups, but were higher or diverged more in the fall group.

**Figure 3:**
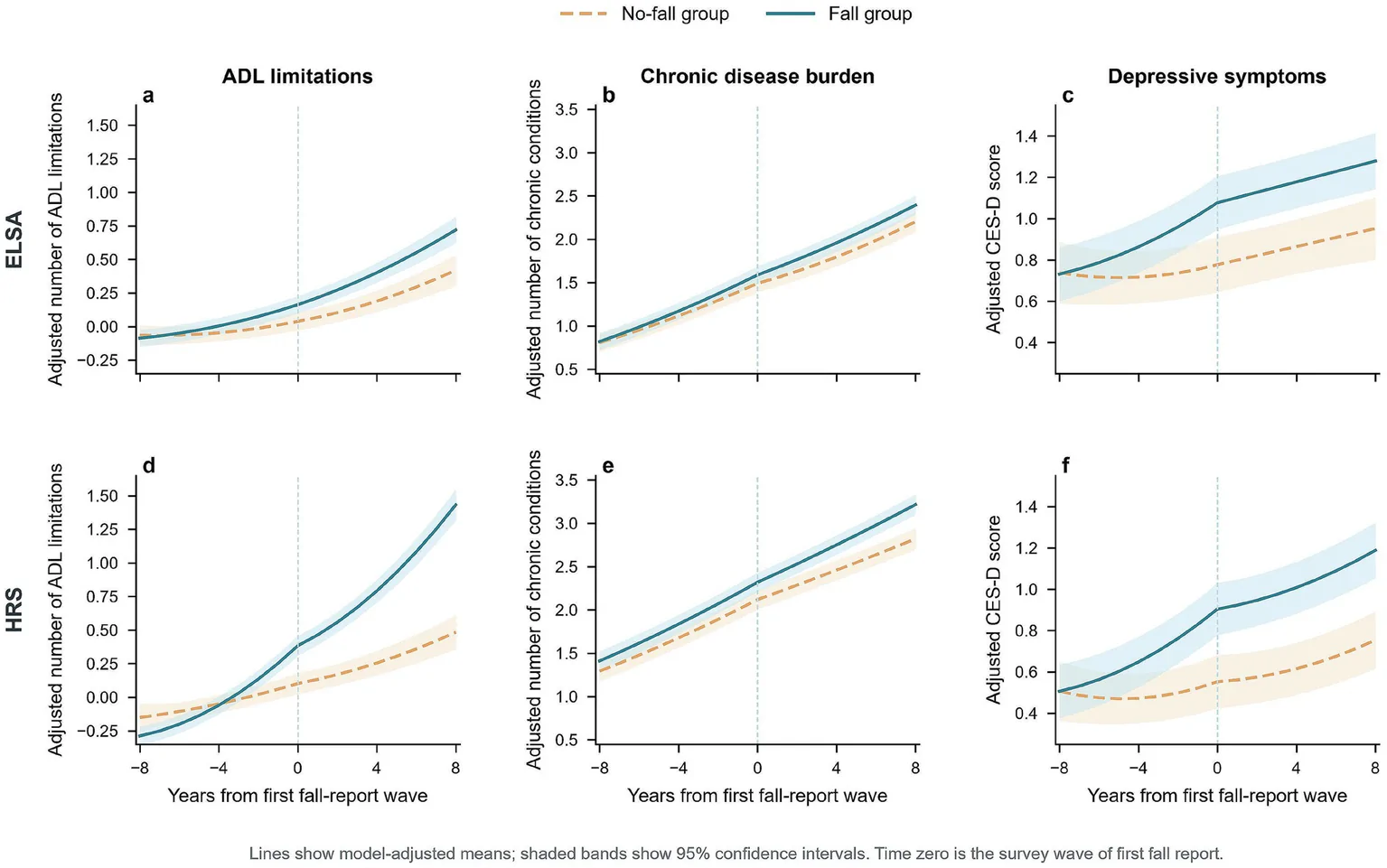
Adjusted trajectories of ADL limitations, chronic disease burden, and depressive symptoms in ELSA **(a–c)** and HRS **(d–f)**. Solid blue lines denote the fall group and dashed orange lines the non-fall group; shaded bands are 95% confidence intervals. Time zero is the first fall-report wave or matched pseudo-index wave.

In ELSA (Figure 3b), the adjusted chronic condition count difference was 0.01 (95% CI, −0.06 to 0.09; *p* = 0.726) at −8 years. At time zero, it was 0.10 (95% CI, 0.02 to 0.18; *p* = 0.017). At +8 years, it was 0.19 (95% CI, 0.08 to 0.30; *p* < 0.001). The corresponding multimorbidity OR was 0.91 (95% CI, 0.73 to 1.15) at −8 years. It was 1.36 (95% CI, 1.16 to 1.58) at time zero. At +8 years, it was 1.56 (95% CI, 1.25 to 1.94).

A similar but more pronounced pattern was observed in HRS (Figure 3e). At −8 years, the adjusted chronic condition count difference was 0.12 (95% CI, 0.03 to 0.21; *p* = 0.011). At time zero, it was 0.20 (95% CI, 0.11 to 0.29; *p* < 0.001). At +8 years, it was 0.40 (95% CI, 0.29 to 0.50; *p* < 0.001). The corresponding multimorbidity OR was 1.30 (95% CI, 1.06 to 1.60) at −8 years. It was 1.39 (95% CI, 1.19 to 1.61) at time zero. At +8 years, it was 2.16 (95% CI, 1.68 to 2.77).

Across cohorts, participants in the fall group had less favorable chronic disease trajectories. Differences in chronic condition count and multimorbidity widened after the first fall-report wave.

### 3.3 Trajectories of depressive symptoms

After adjustment, the fall group had higher depressive symptom scores at the first fall-report wave in both cohorts. These differences remained elevated thereafter.

In ELSA (Figure 3c), the adjusted depressive symptom difference was −0.01 (95% CI, −0.14 to 0.13; *p* = 0.923) at −8 years. At time zero, it was 0.30 (95% CI, 0.19 to 0.41; *p* < 0.001). At +8 years, it was 0.33 (95% CI, 0.19 to 0.46; *p* < 0.001). The corresponding depression OR was 1.03 (95% CI, 0.81 to 1.31) at −8 years. It was 1.51 (95% CI, 1.30 to 1.76) at time zero. At +8 years, it was 1.52 (95% CI, 1.27 to 1.82). The selected quadratic trajectory indicated that the group difference emerged before the report wave and then remained elevated.

Findings in HRS were similar (Figure 3f). At −8 years, the adjusted depressive symptom difference was 0.00 (95% CI, −0.13 to 0.13; *p* = 0.997). At time zero, it was 0.35 (95% CI, 0.26 to 0.45; *p* < 0.001). At +8 years, it was 0.43 (95% CI, 0.31 to 0.56; p < 0.001). The corresponding depression OR was 1.05 (95% CI, 0.83 to 1.33) at −8 years. It was 1.63 (95% CI, 1.41 to 1.89) at time zero. At +8 years, it was 1.62 (95% CI, 1.36 to 1.92). The nonlinear pattern did not support continued post-index acceleration.

Across cohorts, depressive symptom differences emerged before the first fall-report wave and remained elevated thereafter, without consistent post-index acceleration.

### 3.4 Trajectories of ADL limitations

ADL limitations showed the largest and most persistent group differences, although the selected nonlinear forms differed between cohorts.

In ELSA (Figure 3a), the adjusted ADL limitation difference was −0.02 (95% CI, −0.09 to 0.04; *p* = 0.482) at −8 years. At time zero, it was 0.12 (95% CI, 0.07 to 0.18; *p* < 0.001). At +8 years, it was 0.30 (95% CI, 0.17 to 0.43; *p* < 0.001). The corresponding ADL disability OR was 1.09 (95% CI, 0.85 to 1.40) at −8 years. It was 1.61 (95% CI, 1.38 to 1.88) at time zero. At +8 years, it was 1.55 (95% CI, 1.31 to 1.84).

The HRS quadratic model showed an early group difference (Figure 3d). At −8 years, the fall group had fewer ADL limitations (difference = −0.14; 95% CI, −0.23 to −0.04; *p* = 0.005). The trajectories then crossed. The difference was 0.28 (95% CI, 0.21 to 0.35; *p* < 0.001) at time zero. At +8 years, it was 0.95 (95% CI, 0.80 to 1.10; *p* < 0.001). The corresponding ADL disability OR was 0.95 (95% CI, 0.73 to 1.25) at −8 years. It was 2.02 (95% CI, 1.74 to 2.34) at time zero. At +8 years, it was 2.87 (95% CI, 2.44 to 3.38).

Across cohorts, ADL limitation trajectories separated around the first fall-report wave and continued to diverge thereafter. Among the three outcomes, functional limitations showed the most pronounced and consistent association with fall reports.

### 3.5 Subgroup analyses

Subgroup analyses were exploratory. They suggested heterogeneity mainly in ADL limitations and depressive symptoms, but were not interpreted as evidence of causal effect modification.

**Figure 4:**
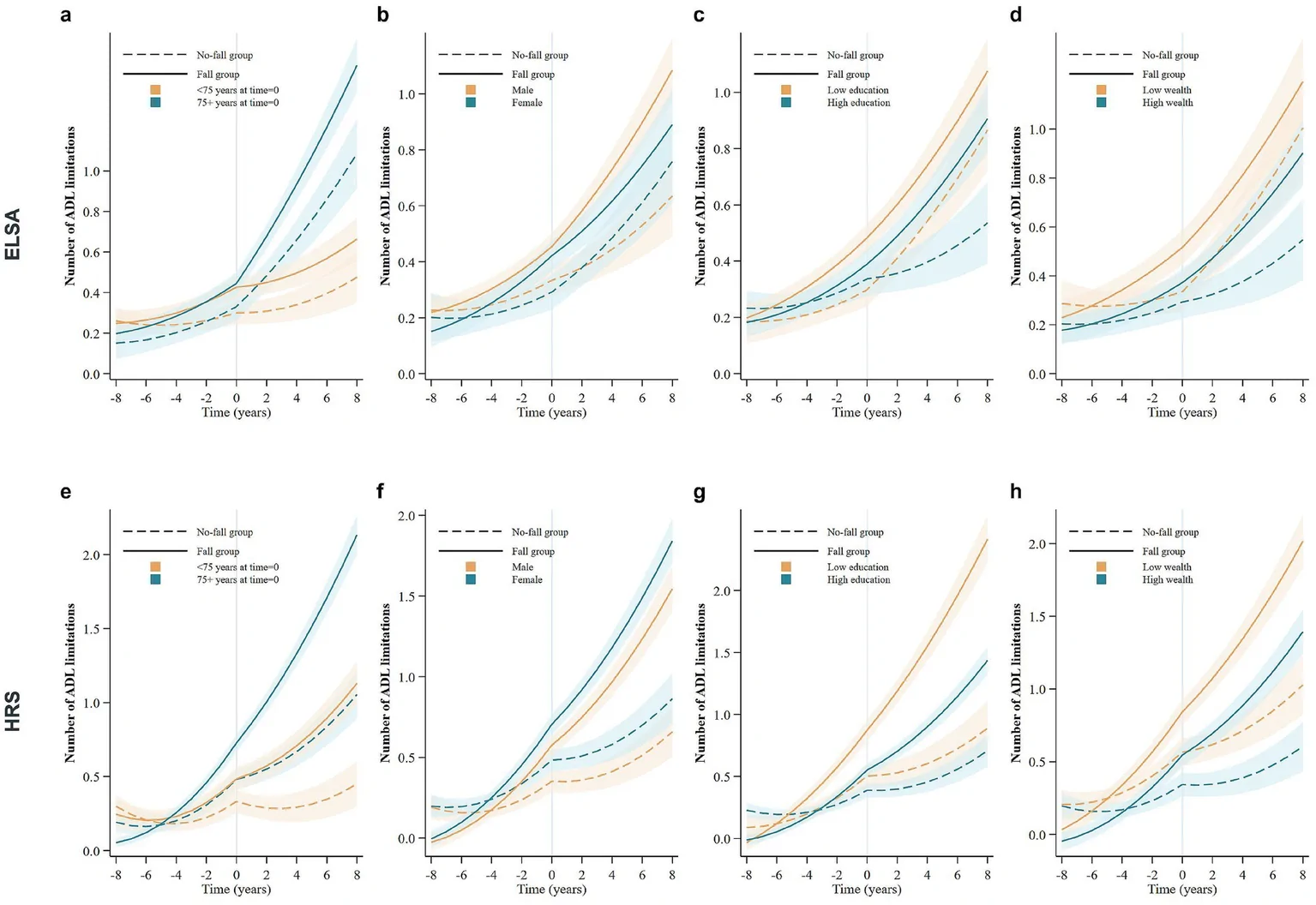
Subgroup-specific ADL trajectories in ELSA **(a–d)** and HRS **(e–h)**, stratified by age, sex, education, and wealth. Solid lines denote the fall group, dashed lines the non-fall group, and shaded bands 95% confidence intervals.

In HRS, ADL trajectory differences were larger among older adults and participants with lower education (Figures 4e–h). At +8 years, the difference-in-differences for age ≥75 versus <75 years was 0.40 limitations (95% CI, 0.11 to 0.69; *p* = 0.007). The corresponding contrast for high versus low education was −0.80 (95% CI, −1.13 to −0.46; *p* < 0.001). Subgroup differences were weaker in ELSA (Figures 4a–d). At +8 years, women had a smaller fall-related ADL difference than men (difference-in-differences = −0.32; 95% CI, −0.58 to −0.05; *p* = 0.019).

**Figure 5:**
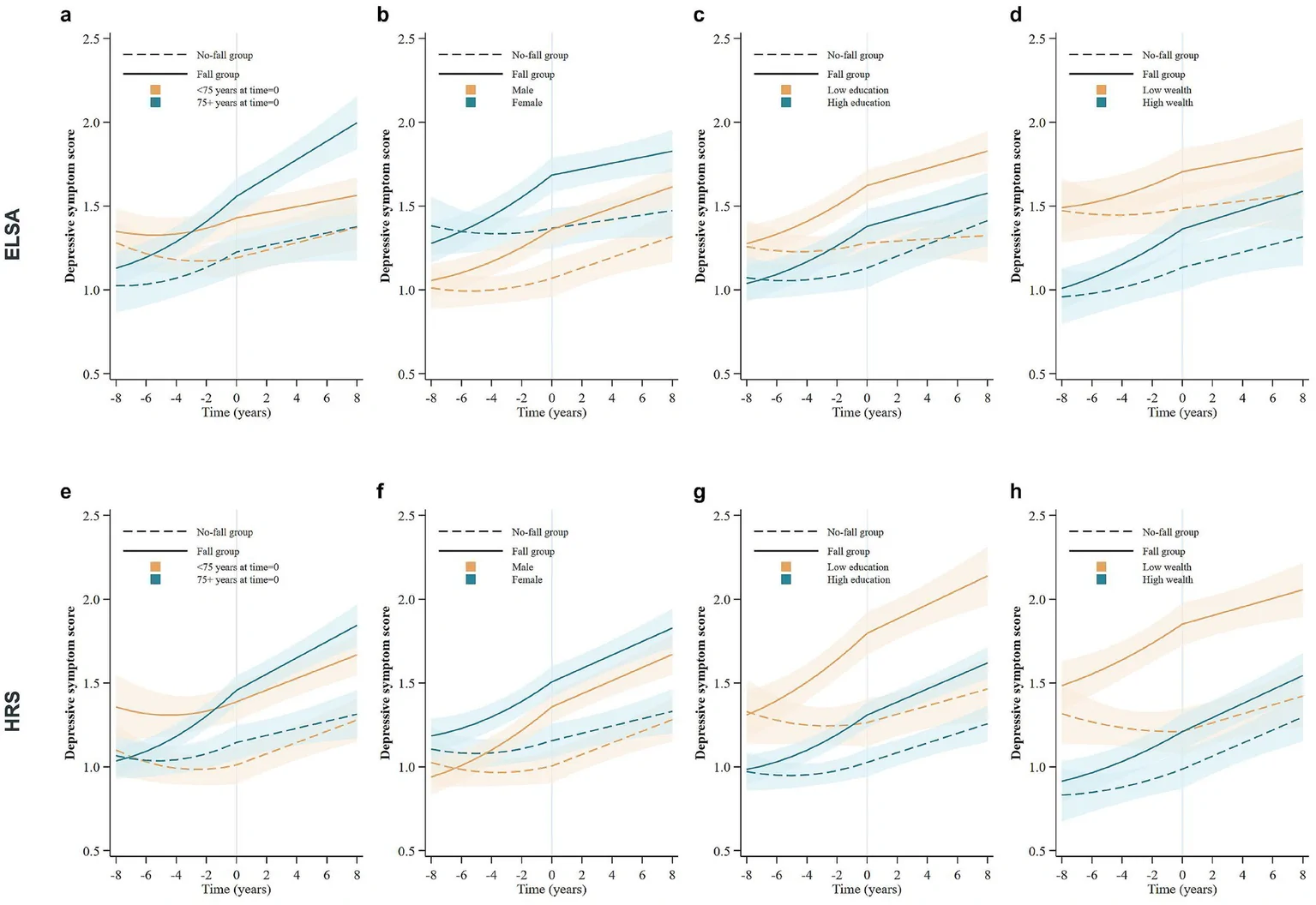
Subgroup-specific depressive symptom trajectories in ELSA **(a–d)** and HRS **(e–h)**, stratified by age, sex, education, and wealth. Solid lines denote the fall group, dashed lines the non-fall group, and shaded bands 95% confidence intervals.

Education and wealth more clearly stratified depressive symptom patterns in HRS (Figures 5e–h). At time zero, the difference-in-differences for high versus low education was −0.25 (95% CI, −0.47 to −0.03; *p* = 0.023). The corresponding contrast for high versus low wealth was −0.42 (95% CI, −0.66 to −0.18; *p* < 0.001). In ELSA (Figures 5a–d), the older group had a larger +8-year difference. For participants aged ≥75 versus <75 years, the contrast was 0.43 (95% CI, 0.13 to 0.73; *p* = 0.005).

**Figure 6:**
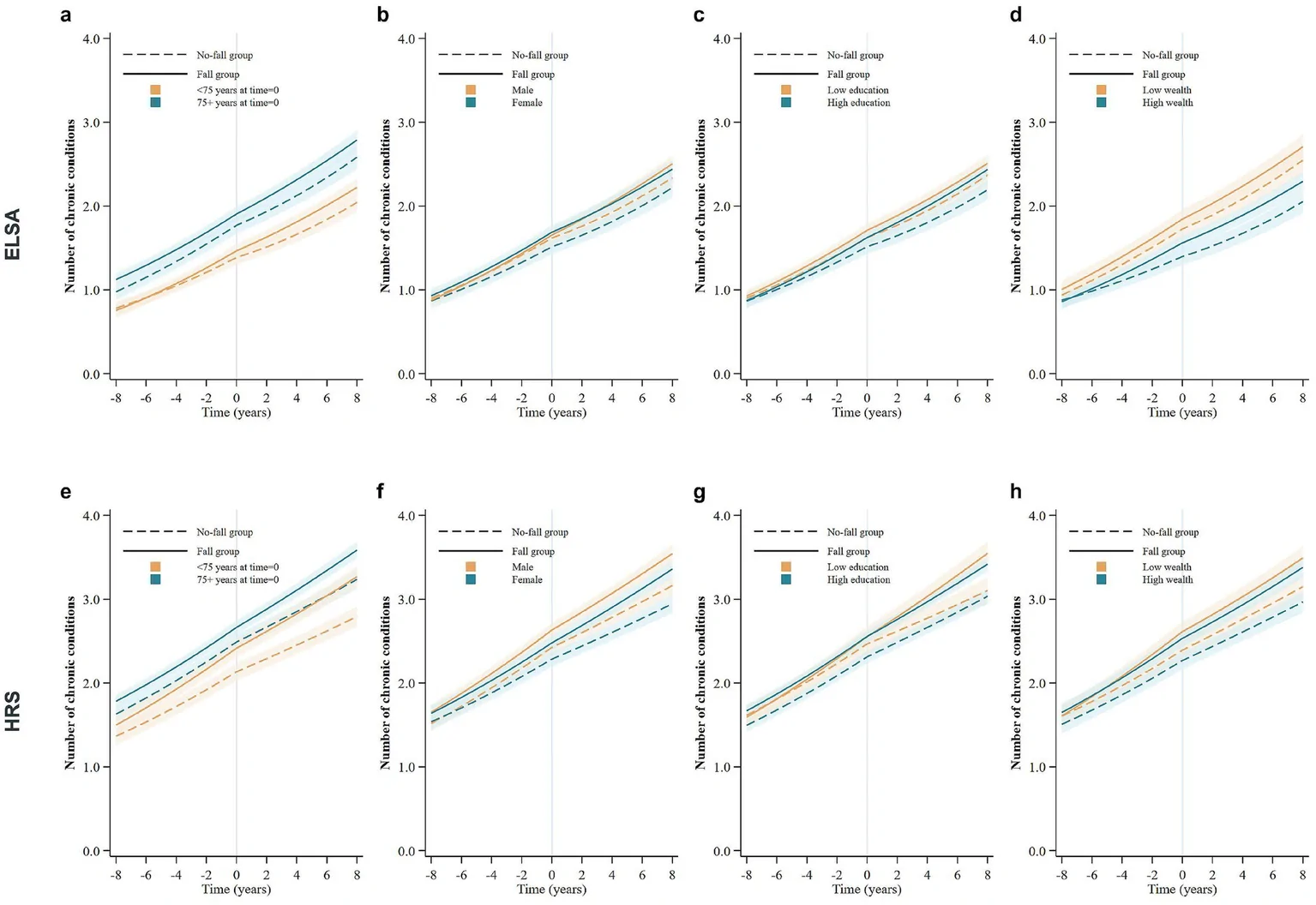
Subgroup-specific chronic disease trajectories in ELSA **(a–d)** and HRS **(e–h)**, stratified by age, sex, education, and wealth. Solid lines denote the fall group, dashed lines the non-fall group, and shaded bands 95% confidence intervals.

Group differences in chronic disease trajectories were generally modest across subgroups in both cohorts (Figure 6). In HRS (Figures 6e–h), participants with high education showed a slower post-index increase than those with low education. In ELSA (Figures 6a–d), age-related differences were small and inconsistent across the principal time points.

Exploratory differences in functional and psychological burden were most apparent among some older or socioeconomically disadvantaged groups, particularly in HRS. Because multiple subgroup contrasts were examined, these patterns require cautious interpretation.

### 3.6 Sensitivity analyses

Restricting the analytic sample to participants with at least three, four, or five waves did not materially alter the principal post-index findings (Supplementary Tables A4–A15 and Supplementary Figures A1–A6). The negative HRS ADL difference at −8 years attenuated with stricter follow-up requirements. It was −0.13 among participants with at least three waves and −0.07 among those with at least four waves. Among participants with at least five waves, it was −0.05 (*p* = 0.087). This attenuation is consistent with a contribution from selective long-term retention.

## 4 Discussion

This matched event-centered study used two large longitudinal aging cohorts to examine nonlinear trajectories around the first fall-report wave. Three findings were consistent across cohorts. First, multidimensional health differences began to emerge before the report wave. Second, chronic disease and especially ADL differences widened after the index wave. Depressive symptom differences remained elevated without consistently accelerating. Third, ADL limitations showed the largest long-term separation. These findings represent event-aligned associations and do not establish that the fall caused the subsequent changes.

Our findings extend previous evidence on fall risk factors and post-fall outcomes by clarifying their temporal pattern. Older age, chronic conditions, depressive symptoms, impaired balance, activity restriction, and polypharmacy are established correlates of fall risk (Montero-Odasso et al., 2022; Ganz and Latham, 2020; Ambrose et al., 2013; Tinetti et al., 1988). Most studies, however, have examined baseline predictors or single post-fall outcomes rather than continuous trajectories spanning the event. Participants who later reported falls in our study already showed less favorable trajectories across all three health domains before the report wave. A prolonged accumulation of vulnerability may therefore precede a first fall report.

The increase in chronic disease burden on both sides of the report wave is consistent with falls occurring against a background of declining physiological reserve and accumulating multimorbidity. Potential pathways linking multimorbidity to falls include reduced muscle strength, fatigue, pain, sensory impairment, balance problems, and lower physical activity (Marengoni et al., 2011; Ryan et al., 2015). Multimorbidity is also accompanied by polypharmacy. Antihypertensives, sedatives, antidepressants, and glucose-lowering drugs may increase fall risk through orthostatic hypotension, dizziness, delayed reactions, or hypoglycaemia (Dhalwani et al., 2017). Group differences in chronic disease burden widened after the report wave, particularly in HRS. Reduced activity, hospitalization, bed rest, or greater medical contact after a fall could further complicate disease management. These processes may reinforce a cycle of multimorbidity, functional decline, falls, and further deterioration.

Depressive symptoms also appeared to form part of pre-fall vulnerability. In both cohorts, the fall group had higher scores at the report wave and remained at higher levels thereafter. Binary analyses likewise showed higher odds of depression. Previous reviews support a close association between depressive symptoms and falls (Kvelde et al., 2013; Iaboni and Flint, 2013). Depression may increase fall risk through impaired attention, sleep disturbance, fatigue, psychomotor slowing, reduced physical activity, and poorer self-care. Conversely, fear of falling, activity avoidance, reduced social participation, and functional dependence may worsen depressive symptoms after a fall (Vellas et al., 1997; Murphy et al., 2002; Thomas et al., 2022). Unlike ADL limitations, depressive symptoms did not consistently accelerate after the report wave but remained elevated. Mental health burden may therefore develop before a first fall report and continue to affect recovery and functional maintenance.

The ADL limitations showed the largest long-term separation, consistent with evidence linking falls and fall-related injuries to subsequent nursing home admission (Tinetti and Williams, 1997) and later ADL impairment (Adam et al., 2024). ADL limitations showed the largest separation, although the early HRS crossover warrants careful interpretation. Age was well balanced after matching (absolute SMD = 0.006), making simple age imbalance an unlikely explanation. The fall group nevertheless had greater baseline morbidity. Moreover, the negative ADL difference at −8 years attenuated when analyses were restricted to participants with longer follow-up. This pattern is compatible with selective survival or retention rather than a protective pre-fall state. Differences in item wording, interview administration, and reporting may also remain despite harmonization of the six ADL domains. We therefore regard the crossover as cohort- and selection-sensitive, not as evidence that future fallers initially had better function.

Socioeconomic patterns were consistent with social determinants of health, cumulative inequality, and social stratification perspectives. The World Health Organization framework emphasizes that education, material resources, housing, and access to care shape health inequalities (World Health Organization, 2025). Cumulative inequality theory further proposes that disadvantages and protective resources accumulate across the life course, shaping both health risks and recovery capacity (Ferraro and Shippee, 2009). Education may influence health literacy, care navigation, and the ability to act on rehabilitation advice. Wealth may buffer transport costs, home modifications, informal care needs, and interruptions in paid support. Recent evidence also links lower socioeconomic position to less favorable outcomes after severe falls (Speckmann et al., 2025). Our subgroup findings are compatible with these pathways but do not test them directly.

National context may influence how vulnerability translates into recovery. England’s universal NHS reduces insurance-related barriers, although waiting times and local service capacity may constrain rehabilitation. In the United States, heterogeneous coverage, cost sharing, and fragmented delivery may produce wider variation in access to post-fall care (Anderson et al., 2022; Rice et al., 2020). These features could contribute to cross-cohort differences, including the larger HRS ADL separation. However, this two-cohort design cannot distinguish health-system effects from differences in age structure, morbidity, selection, or measurement.

Fall prevention and post-fall management should therefore be integrated into broader geriatric care rather than confined to injury avoidance. A first fall report could trigger medication review, chronic disease management, mental health screening, functional assessment, progressive rehabilitation, and home-safety evaluation. The exploratory subgroup findings suggest particular attention to older adults and those with lower education or wealth. For these groups, services may require proactive referral, care navigation, transport or home-based rehabilitation, and strengthened social support rather than passive signposting. Exercise and multifactorial fall-prevention programs provide evidence-based components of this broader approach (Sherrington et al., 2019; Hopewell et al., 2018).

This study has several strengths. Two independent aging cohorts with long-term repeated measurements allowed us to assess whether patterns were reproducible across population settings. The matched event-centered design provided a comparable non-fall reference group, separating general aging trends from differences aligned with first fall reports. Simultaneous assessment of chronic disease burden, depressive symptoms, and ADL limitations captured complementary dimensions of health. Reporting both continuous trajectories and binary outcomes also improved clinical interpretability.

This study also has several limitations. First, time zero represented the wave of the first fall report rather than the exact fall date. With approximately two-year intervals, gradual pre-fall deterioration and acute post-fall change may have occurred within the same observation interval. The analysis therefore cannot separate immediate causal effects from longer-term event-aligned trends. Second, falls, chronic conditions, depressive symptoms, and ADL limitations were largely self-reported. Harmonization cannot eliminate all differences in wording or administration between cohorts. Third, CEM achieved excellent balance on matching variables, but residual HRS differences remained in arthritis, chronic condition count, and multimorbidity. Unmeasured confounding by medication use, gait, strength, balance, pain, cognition, home environment, or social support is also possible. Fourth, exclusion because of missingness, insufficient follow-up, or lack of a match may have introduced selection and survivorship bias. The attenuation of the early HRS ADL difference in longer-follow-up samples supports this concern. Finally, the study was not designed to estimate causal effects of national health-system differences. The subgroup analyses were exploratory and involved multiple comparisons.

## 5 Conclusion

Across ELSA and HRS, adults who later reported a fall showed less favorable long-term trajectories of chronic disease burden, depressive symptoms, and ADL limitations around the first fall-report wave. Functional differences widened most markedly after the index wave. Because the exact fall date was unavailable, these findings identify a period of multidimensional vulnerability rather than an acute causal effect. A first fall report could therefore trigger sustained, resource-sensitive geriatric assessment and rehabilitation, especially for older adults and those with fewer socioeconomic resources.

## Data Availability

The original contributions presented in the study are included in the article/Supplementary material, further inquiries can be directed to the corresponding authors.
