# Supplementary material for: Trajectories of chronic disease burden, depressive symptoms, and functional limitations related to falls in middle-aged and older adults: a matched longitudinal study
Source: Front Aging Neurosci. 2026 Aug 18;18:1896362. doi: 10.3389/fnagi.2026.1896362 (PMC13529771; doi:10.3389/fnagi.2026.1896362)
Supplement: Supplementary file 1 [file Data_Sheet_1.pdf]

Supplementary material

Contents

Methods A1. Derivation of population-average odds ratio.
Table A1. Covariate balance before and after coarsened exact matching.
Table A2. Fit comparison between piecewise linear and selected outcome-specific quadratic mixed-effects models.
Table A3. Baseline prevalence of individual diagnoses in the matched ELSA and HRS cohorts.
Table A4. ELSA outcome differences, at least 3 follow-up waves.
Table A5. ELSA trajectory model parameters, at least 3 follow-up waves.
Table A6. ELSA outcome differences, at least 4 follow-up waves.
Table A7. ELSA trajectory model parameters, at least 4 follow-up waves.
Table A8. ELSA outcome differences, at least 5 follow-up waves.
Table A9. ELSA trajectory model parameters, at least 5 follow-up waves.
Table A10. HRS outcome differences, at least 3 follow-up waves.
Table A11. HRS trajectory model parameters, at least 3 follow-up waves.
Table A12. HRS outcome differences, at least 4 follow-up waves.
Table A13. HRS trajectory model parameters, at least 4 follow-up waves.
Table A14. HRS outcome differences, at least 5 follow-up waves.
Table A15. HRS trajectory model parameters, at least 5 follow-up waves.
Figure A1. ELSA trajectories, at least 3 follow-up waves.
Figure A2. ELSA trajectories, at least 4 follow-up waves.
Figure A3. ELSA trajectories, at least 5 follow-up waves.
Figure A4. HRS trajectories, at least 3 follow-up waves.
Figure A5. HRS trajectories, at least 4 follow-up waves.
Figure A6. HRS trajectories, at least 5 follow-up waves.
References.

Methods A1. Derivation of population-average odds ratio.

For the dichotomous outcomes, logistic mixed-effects models with random intercepts yield subject-specific regression coefficients. To aid interpretation at the population level, we derived population-average coefficients from the estimated subject-specific coefficients using the marginalisation approximation described by Hedeker et al. [1].

$$\beta_{PA} = \beta_{SS} / \sqrt{(\sigma_v^2 + \pi^2/3) / (\pi^2/3)}$$

where β_{SS} denotes the subject-specific coefficient and σ_v^2 denotes the estimated variance of the random intercept. Population-average odds ratios were then obtained by exponentiating the population-average coefficients. These odds ratios were reported with the non-fall group as the reference at −8 years, time zero, and +8 years relative to the first fall-report or matched pseudo-index wave.

Supplementary tables

Table A1. Covariate balance before and after coarsened exact matching. SMD denotes the standardised mean difference; VR denotes the variance ratio (fall group/non-fall group). Values near 0 for SMD and near 1 for VR indicate better balance.

Cohort	Covariate	Level	Matched	SMD before	VR before	SMD after	VR after
ELSA	Age (years)	—	Yes	0.038	0.982	-0.004	0.999
ELSA	Birth year	—	Yes	-0.065	0.925	-0.002	0.996
ELSA	Number of chronic conditions	—	No	0.022	1.032	0.018	1.064
ELSA	Number of ADL limitations	—	No	0.053	1.013	0.058	1.013
ELSA	Depressive symptom score	—	No	0.065	0.982	0.046	0.935
ELSA	Sex	Male	Yes	-0.236	1.008	0.000	1.000
ELSA	Sex	Female	Yes	0.236	1.008	0.000	1.000
ELSA	Education level	Low	Yes	-0.043	1.005	0.000	1.000
ELSA	Education level	Intermediate	Yes	0.013	1.007	0.000	1.000
ELSA	Education level	High	Yes	0.050	1.139	0.000	1.000
ELSA	Wealth tertile	Low	No	-0.122	0.885	-0.094	0.912
ELSA	Wealth tertile	Intermediate	No	-0.004	0.997	-0.011	0.993
ELSA	Wealth tertile	High	No	0.117	1.060	0.098	1.054
ELSA	Weekly moderate-to-vigorous physical activity	Yes	No	0.028	0.965	0.033	0.959
ELSA	Alcohol consumption	Yes	No	-0.033	1.081	-0.005	1.013
ELSA	Current smoking	Yes	No	-0.097	0.814	-0.070	0.866
ELSA	Hypertension	Yes	No	-0.021	0.990	-0.045	0.977
ELSA	Diabetes	Yes	No	-0.012	0.962	0.010	1.032
ELSA	Cancer	Yes	No	0.044	1.163	0.030	1.111
ELSA	Chronic lung disease	Yes	No	-0.085	0.702	-0.083	0.714
ELSA	Heart disease	Yes	No	-0.056	0.904	-0.045	0.923
ELSA	Stroke	Yes	No	0.009	1.048	0.038	1.208
ELSA	Psychiatric condition	Yes	No	0.055	1.297	0.075	1.442
ELSA	Arthritis	Yes	No	0.113	1.102	0.095	1.088
ELSA	Multimorbidity	Yes	No	0.020	1.016	0.011	1.009
ELSA	ADL disability	Yes	No	0.074	1.148	0.085	1.169
ELSA	Depression	Yes	No	0.059	1.101	0.044	1.073
HRS	Age (years)	—	Yes	0.095	0.993	-0.006	1.023
HRS	Birth year	—	Yes	-0.146	0.905	-0.003	1.017
HRS	Number of chronic conditions	—	No	0.096	0.994	0.111	0.979
HRS	Number of ADL limitations	—	No	0.013	0.820	0.021	0.868
HRS	Depressive symptom score	—	No	0.108	1.232	0.097	1.206
HRS	Sex	Male	Yes	-0.176	0.971	0.000	1.000
HRS	Sex	Female	Yes	0.176	0.971	0.000	1.000
HRS	Education level	Low	Yes	-0.066	0.932	0.000	1.000
HRS	Education level	Intermediate	Yes	0.045	0.990	0.000	1.000
HRS	Education level	High	Yes	0.019	1.032	0.000	1.000
HRS	Wealth tertile	Low	No	-0.082	0.923	-0.025	0.977
HRS	Wealth tertile	Intermediate	No	0.055	1.041	0.063	1.046
HRS	Wealth tertile	High	No	0.022	1.010	-0.037	0.982
HRS	Weekly moderate-to-vigorous physical activity	Yes	No	0.008	0.991	0.028	0.970
HRS	Alcohol consumption	Yes	No	-0.023	0.996	-0.022	0.996
HRS	Current smoking	Yes	No	-0.086	0.803	-0.067	0.846
HRS	Hypertension	Yes	No	-0.034	1.008	-0.016	1.004
HRS	Diabetes	Yes	No	0.051	1.095	0.086	1.163
HRS	Cancer	Yes	No	0.041	1.085	0.038	1.078
HRS	Chronic lung disease	Yes	No	-0.047	0.865	-0.053	0.851
HRS	Heart disease	Yes	No	0.047	1.060	0.060	1.076
HRS	Stroke	Yes	No	0.001	1.005	0.007	1.025
HRS	Psychiatric condition	Yes	No	0.107	1.408	0.085	1.327
HRS	Arthritis	Yes	No	0.145	0.964	0.142	0.964
HRS	Multimorbidity	Yes	No	0.098	0.965	0.110	0.960

Cohort	Covariate	Level	Matched	SMD before	VR before	SMD after	VR after
HRS	ADL disability	Yes	No	0.071	1.192	0.070	1.192
HRS	Depression	Yes	No	0.100	1.191	0.091	1.177

Table A2. Fit comparison between piecewise linear and selected outcome-specific quadratic mixed-effects models. LRT values are likelihood-ratio χ^2 statistics with degrees of freedom in parentheses. Models were estimated by maximum likelihood for comparison.

Cohort	Outcome	Linear AIC	Linear BIC	Selected AIC	Selected BIC	LRT χ^2 (df)	p
ELSA	ADL limitations	47,660.77	47,905.61	47,612.10	47,872.74	52.67 (2)	<0.001
ELSA	Chronic conditions	34,942.91	35,124.57	34,812.22	35,017.58	136.69 (3)	<0.001
ELSA	Depressive symptoms	69,133.30	69,376.99	69,125.78	69,377.33	9.52 (1)	0.002
HRS	ADL limitations	62,024.92	62,275.25	61,814.60	62,097.22	218.33 (4)	<0.001
HRS	Chronic conditions	43,750.65	43,936.40	43,724.17	43,934.14	32.49 (3)	<0.001
HRS	Depressive symptoms	78,589.34	78,837.72	78,576.34	78,840.74	17.00 (2)	<0.001

Selected quadratic terms were: ELSA ADL, common pre- and post-index terms; ELSA chronic conditions, common pre- and post-index terms plus a group-specific post-index term; ELSA depressive symptoms, a common pre-index term; HRS ADL, common and group-specific pre- and post-index terms; HRS chronic conditions, common pre- and post-index terms plus a group-specific post-index term; and HRS depressive symptoms, common pre- and post-index terms. AIC and the LRT supported the selected forms; BIC was slightly lower for the linear depressive-symptom models, so interpretation emphasised the overall evidence and parsimony.

Table A3. Baseline prevalence of individual diagnoses in the matched ELSA and HRS cohorts. Values are n (%); |SMD| denotes the absolute standardised mean difference.

Diagnosis	ELSA non-fall	ELSA fall	ELSA |SMD|	HRS non-fall	HRS fall	HRS |SMD|
Hypertension	689 (38.5)	650 (36.3)	0.045	1,186 (56.8)	1,169 (56.0)	0.016
Diabetes	144 (8.0)	149 (8.3)	0.010	325 (15.6)	393 (18.8)	0.086
Cancer	109 (6.1)	122 (6.8)	0.030	293 (14.0)	321 (15.4)	0.038
Chronic lung disease	110 (6.1)	77 (4.3)	0.083	185 (8.9)	155 (7.4)	0.053
Heart disease	316 (17.6)	286 (16.0)	0.045	470 (22.5)	523 (25.0)	0.060
Stroke	60 (3.4)	73 (4.1)	0.038	147 (7.0)	151 (7.2)	0.007
Psychiatric condition	56 (3.1)	82 (4.6)	0.075	128 (6.1)	174 (8.3)	0.085
Arthritis	496 (27.7)	574 (32.0)	0.095	1,104 (52.8)	1,251 (59.9)	0.142

ELSA sensitivity analyses

Table A4. Differences in outcomes between fall and non-fall groups when analyses are restricted to ELSA participants with at least 3 follow-up waves.

Outcome	Years from index wave	Diff / OR (95% CI)	P-value
Number of ADL limitations	-8	-0.02 (-0.08 to 0.05)	0.611
	0	0.13 (0.07 to 0.18)	<0.001
	8	0.32 (0.20 to 0.45)	<0.001
Number of chronic conditions	-8	0.05 (-0.04 to 0.14)	0.294
	0	0.14 (0.04 to 0.23)	0.004
	8	0.23 (0.12 to 0.35)	<0.001
Depressive symptom score	-8	0.00 (-0.14 to 0.15)	0.984
	0	0.29 (0.18 to 0.40)	<0.001
	8	0.31 (0.16 to 0.45)	<0.001
ADL disability	-8	1.14 (0.85 to 1.51)	0.381
	0	1.69 (1.42 to 2.02)	<0.001
	8	1.62 (1.35 to 1.95)	<0.001
Multimorbidity	-8	0.97 (0.75 to 1.26)	0.815
	0	1.37 (1.15 to 1.63)	<0.001
	8	1.93 (1.53 to 2.44)	<0.001
Depression	-8	1.06 (0.81 to 1.38)	0.695
	0	1.52 (1.28 to 1.80)	<0.001
	8	1.54 (1.26 to 1.86)	<0.001

Values are model-based differences (fall group minus non-fall group) for continuous outcomes or odds ratios for dichotomous outcomes, with 95% confidence intervals. Models are adjusted for baseline covariates as described in the main text.

Table A5. Model parameters for fall-related trajectory differences when analyses are restricted to ELSA participants with at least 3 follow-up waves.

Outcome	Parameter	β (95% CI)	P-value
Number of ADL limitations	Difference at time zero (t=0)	0.13 (0.07 to 0.18)	<0.001
	Pre-index linear slope difference	0.02 (0.01 to 0.03)	<0.001
	Post-index linear slope difference	0.02 (0.01 to 0.04)	0.003
Number of chronic conditions	Difference at time zero (t=0)	0.14 (0.04 to 0.23)	0.004
	Pre-index linear slope difference	0.01 (0.00 to 0.02)	0.046
	Post-index linear slope difference	0.02 (0.01 to 0.03)	0.007
	Post-index quadratic slope difference	-0.00 (-0.00 to 0.00)	0.088
Depressive symptom score	Difference at time zero (t=0)	0.29 (0.18 to 0.40)	<0.001
	Pre-index linear slope difference	0.04 (0.01 to 0.06)	0.001
	Post-index linear slope difference	0.00 (-0.02 to 0.02)	0.808

Parameters represent between-group differences at time zero and differences in pre-index and post-index slopes. Time zero refers to the first fall-report wave in the fall group or the corresponding pseudo-index wave in the non-fall group.

Table A6. Differences in outcomes between fall and non-fall groups when analyses are restricted to ELSA participants with at least 4 follow-up waves.

Outcome	Years from index wave	Diff / OR (95% CI)	P-value
Number of ADL limitations	-8	0.01 (-0.05 to 0.08)	0.704
	0	0.14 (0.07 to 0.20)	<0.001
	8	0.37 (0.25 to 0.50)	<0.001
Number of chronic conditions	-8	0.07 (-0.02 to 0.17)	0.141
	0	0.17 (0.06 to 0.27)	0.002
	8	0.34 (0.20 to 0.47)	<0.001
Depressive symptom score	-8	0.14 (-0.01 to 0.29)	0.065
	0	0.33 (0.21 to 0.46)	<0.001
	8	0.40 (0.25 to 0.55)	<0.001
ADL disability	-8	1.18 (0.87 to 1.61)	0.291
	0	1.83 (1.50 to 2.24)	<0.001
	8	1.83 (1.49 to 2.25)	<0.001
Multimorbidity	-8	1.08 (0.79 to 1.46)	0.633
	0	1.47 (1.20 to 1.79)	<0.001
	8	1.88 (1.46 to 2.43)	<0.001
Depression	-8	1.35 (1.00 to 1.81)	0.048
	0	1.61 (1.33 to 1.97)	<0.001
	8	1.66 (1.34 to 2.04)	<0.001

Values are model-based differences (fall group minus non-fall group) for continuous outcomes or odds ratios for dichotomous outcomes, with 95% confidence intervals. Models are adjusted for baseline covariates as described in the main text.

Table A7. Model parameters for fall-related trajectory differences when analyses are restricted to ELSA participants with at least 4 follow-up waves.

Outcome	Parameter	β (95% CI)	P-value
Number of ADL limitations	Difference at time zero (t=0)	0.14 (0.07 to 0.20)	<0.001
	Pre-index linear slope difference	0.02 (0.00 to 0.03)	0.004
	Post-index linear slope difference	0.03 (0.01 to 0.05)	<0.001
Number of chronic conditions	Difference at time zero (t=0)	0.17 (0.06 to 0.27)	0.002
	Pre-index linear slope difference	0.01 (-0.00 to 0.02)	0.059
	Post-index linear slope difference	0.03 (0.01 to 0.05)	<0.001
	Post-index quadratic slope difference	-0.00 (-0.00 to 0.00)	0.061
Depressive symptom score	Difference at time zero (t=0)	0.33 (0.21 to 0.46)	<0.001
	Pre-index linear slope difference	0.02 (0.00 to 0.05)	0.036
	Post-index linear slope difference	0.01 (-0.01 to 0.03)	0.403

Parameters represent between-group differences at time zero and differences in pre-index and post-index slopes. Time zero refers to the first fall-report wave in the fall group or the corresponding pseudo-index wave in the non-fall group.

Table A8. Differences in outcomes between fall and non-fall groups when analyses are restricted to ELSA participants with at least 5 follow-up waves.

Outcome	Years from index wave	Diff / OR (95% CI)	P-value
Number of ADL limitations	-8	-0.01 (-0.08 to 0.06)	0.764
	0	0.11 (0.05 to 0.17)	<0.001
	8	0.25 (0.13 to 0.38)	<0.001
Number of chronic conditions	-8	0.11 (0.01 to 0.21)	0.038
	0	0.17 (0.05 to 0.29)	0.006
	8	0.25 (0.11 to 0.40)	<0.001
Depressive symptom score	-8	0.21 (0.06 to 0.36)	0.008
	0	0.27 (0.14 to 0.41)	<0.001
	8	0.35 (0.19 to 0.51)	<0.001
ADL disability	-8	1.01 (0.74 to 1.38)	0.947
	0	1.72 (1.37 to 2.16)	<0.001
	8	1.65 (1.31 to 2.07)	<0.001
Multimorbidity	-8	1.10 (0.79 to 1.53)	0.562
	0	1.50 (1.19 to 1.89)	<0.001
	8	1.95 (1.49 to 2.56)	<0.001
Depression	-8	1.56 (1.15 to 2.12)	0.005
	0	1.53 (1.23 to 1.91)	<0.001
	8	1.56 (1.23 to 1.96)	<0.001

Values are model-based differences (fall group minus non-fall group) for continuous outcomes or odds ratios for dichotomous outcomes, with 95% confidence intervals. Models are adjusted for baseline covariates as described in the main text.

Table A9. Model parameters for fall-related trajectory differences when analyses are restricted to ELSA participants with at least 5 follow-up waves.

Outcome	Parameter	β (95% CI)	P-value
Number of ADL limitations	Difference at time zero (t=0)	0.11 (0.05 to 0.17)	<0.001
	Pre-index linear slope difference	0.02 (0.01 to 0.02)	0.002
	Post-index linear slope difference	0.02 (0.00 to 0.03)	0.035
Number of chronic conditions	Difference at time zero (t=0)	0.17 (0.05 to 0.29)	0.006
	Pre-index linear slope difference	0.01 (-0.01 to 0.02)	0.267
	Post-index linear slope difference	0.02 (0.00 to 0.04)	0.024
	Post-index quadratic slope difference	-0.00 (-0.00 to 0.00)	0.081
Depressive symptom score	Difference at time zero (t=0)	0.27 (0.14 to 0.41)	<0.001
	Pre-index linear slope difference	0.01 (-0.01 to 0.03)	0.486
	Post-index linear slope difference	0.01 (-0.01 to 0.03)	0.350

Parameters represent between-group differences at time zero and differences in pre-index and post-index slopes. Time zero refers to the first fall-report wave in the fall group or the corresponding pseudo-index wave in the non-fall group.

HRS sensitivity analyses

Table A10. Differences in outcomes between fall and non-fall groups when analyses are restricted to HRS participants with at least 3 follow-up waves.

Outcome	Years from index wave	Diff / OR (95% CI)	P-value
Number of ADL limitations	-8	-0.13 (-0.20 to -0.07)	<0.001
	0	0.21 (0.15 to 0.27)	<0.001
	8	0.83 (0.68 to 0.98)	<0.001
Number of chronic conditions	-8	0.15 (0.05 to 0.24)	0.003
	0	0.26 (0.17 to 0.36)	<0.001
	8	0.41 (0.30 to 0.53)	<0.001
Depressive symptom score	-8	0.09 (-0.04 to 0.22)	0.180
	0	0.39 (0.29 to 0.50)	<0.001
	8	0.41 (0.28 to 0.53)	<0.001
ADL disability	-8	1.17 (0.86 to 1.58)	0.312
	0	1.94 (1.65 to 2.28)	<0.001
	8	2.54 (2.14 to 3.01)	<0.001
Multimorbidity	-8	1.43 (1.14 to 1.80)	0.002
	0	1.43 (1.21 to 1.68)	<0.001
	8	1.73 (1.35 to 2.22)	<0.001
Depression	-8	1.20 (0.92 to 1.56)	0.172
	0	1.62 (1.38 to 1.90)	<0.001
	8	1.59 (1.33 to 1.90)	<0.001

Values are model-based differences (fall group minus non-fall group) for continuous outcomes or odds ratios for dichotomous outcomes, with 95% confidence intervals. Models are adjusted for baseline covariates as described in the main text.

Table A11. Model parameters for fall-related trajectory differences when analyses are restricted to HRS participants with at least 3 follow-up waves.

Outcome	Parameter	β (95% CI)	P-value
Number of ADL limitations	Difference at time zero (t=0)	0.21 (0.15 to 0.27)	<0.001
	Pre-index linear slope difference	0.04 (0.03 to 0.05)	<0.001
	Post-index linear slope difference	0.08 (0.06 to 0.10)	<0.001
Number of chronic conditions	Difference at time zero (t=0)	0.26 (0.17 to 0.36)	<0.001
	Pre-index linear slope difference	0.01 (0.00 to 0.03)	0.011
	Post-index linear slope difference	0.02 (0.00 to 0.03)	0.022
	Post-index quadratic slope difference	0.00 (-0.00 to 0.00)	0.580
Depressive symptom score	Difference at time zero (t=0)	0.39 (0.29 to 0.50)	<0.001
	Pre-index linear slope difference	0.04 (0.02 to 0.06)	<0.001
	Post-index linear slope difference	0.00 (-0.01 to 0.02)	0.850

Parameters represent between-group differences at time zero and differences in pre-index and post-index slopes. Time zero refers to the first fall-report wave in the fall group or the corresponding pseudo-index wave in the non-fall group.

Table A12. Differences in outcomes between fall and non-fall groups when analyses are restricted to HRS participants with at least 4 follow-up waves.

Outcome	Years from index wave	Diff / OR (95% CI)	P-value
Number of ADL limitations	-8	-0.07 (-0.14 to -0.01)	0.015
	0	0.26 (0.19 to 0.33)	<0.001
	8	0.83 (0.67 to 0.98)	<0.001
Number of chronic conditions	-8	0.23 (0.13 to 0.34)	<0.001
	0	0.35 (0.25 to 0.45)	<0.001
	8	0.53 (0.41 to 0.66)	<0.001
Depressive symptom score	-8	0.11 (-0.03 to 0.24)	0.111
	0	0.37 (0.26 to 0.47)	<0.001
	8	0.41 (0.27 to 0.56)	<0.001
ADL disability	-8	1.27 (0.92 to 1.75)	0.149
	0	2.30 (1.92 to 2.76)	<0.001
	8	2.80 (2.32 to 3.36)	<0.001
Multimorbidity	-8	1.49 (1.18 to 1.89)	0.001
	0	1.39 (1.17 to 1.67)	<0.001
	8	1.95 (1.49 to 2.56)	<0.001
Depression	-8	1.32 (1.01 to 1.73)	0.045
	0	1.68 (1.41 to 2.00)	<0.001
	8	1.59 (1.31 to 1.93)	<0.001

Values are model-based differences (fall group minus non-fall group) for continuous outcomes or odds ratios for dichotomous outcomes, with 95% confidence intervals. Models are adjusted for baseline covariates as described in the main text.

Table A13. Model parameters for fall-related trajectory differences when analyses are restricted to HRS participants with at least 4 follow-up waves.

Outcome	Parameter	β (95% CI)	P-value
Number of ADL limitations	Difference at time zero (t=0)	0.26 (0.19 to 0.33)	<0.001
	Pre-index linear slope difference	0.04 (0.03 to 0.05)	<0.001
	Post-index linear slope difference	0.07 (0.05 to 0.09)	<0.001
Number of chronic conditions	Difference at time zero (t=0)	0.35 (0.25 to 0.45)	<0.001
	Pre-index linear slope difference	0.01 (0.00 to 0.03)	0.016
	Post-index linear slope difference	0.02 (0.01 to 0.04)	0.003
	Post-index quadratic slope difference	-0.00 (-0.00 to 0.00)	0.985
Depressive symptom score	Difference at time zero (t=0)	0.37 (0.26 to 0.47)	<0.001
	Pre-index linear slope difference	0.03 (0.01 to 0.05)	0.001
	Post-index linear slope difference	0.01 (-0.01 to 0.02)	0.500

Parameters represent between-group differences at time zero and differences in pre-index and post-index slopes. Time zero refers to the first fall-report wave in the fall group or the corresponding pseudo-index wave in the non-fall group.

Table A14. Differences in outcomes between fall and non-fall groups when analyses are restricted to HRS participants with at least 5 follow-up waves.

Outcome	Years from index wave	Diff / OR (95% CI)	P-value
Number of ADL limitations	-8	-0.05 (-0.11 to 0.01)	0.087
	0	0.20 (0.13 to 0.26)	<0.001
	8	0.75 (0.60 to 0.91)	<0.001
Number of chronic conditions	-8	0.26 (0.15 to 0.38)	<0.001
	0	0.40 (0.28 to 0.51)	<0.001
	8	0.56 (0.43 to 0.69)	<0.001
Depressive symptom score	-8	0.08 (-0.06 to 0.21)	0.275
	0	0.35 (0.23 to 0.47)	<0.001
	8	0.37 (0.23 to 0.51)	<0.001
ADL disability	-8	1.42 (1.01 to 2.02)	0.046
	0	2.37 (1.94 to 2.90)	<0.001
	8	2.85 (2.35 to 3.47)	<0.001
Multimorbidity	-8	1.72 (1.33 to 2.21)	<0.001
	0	1.64 (1.35 to 1.99)	<0.001
	8	2.02 (1.52 to 2.69)	<0.001
Depression	-8	1.13 (0.84 to 1.50)	0.417
	0	1.62 (1.34 to 1.96)	<0.001
	8	1.52 (1.24 to 1.85)	<0.001

Values are model-based differences (fall group minus non-fall group) for continuous outcomes or odds ratios for dichotomous outcomes, with 95% confidence intervals. Models are adjusted for baseline covariates as described in the main text.

Table A15. Model parameters for fall-related trajectory differences when analyses are restricted to HRS participants with at least 5 follow-up waves.

Outcome	Parameter	β (95% CI)	P-value
Number of ADL limitations	Difference at time zero (t=0)	0.20 (0.13 to 0.26)	<0.001
	Pre-index linear slope difference	0.03 (0.02 to 0.04)	<0.001
	Post-index linear slope difference	0.07 (0.05 to 0.09)	<0.001
Number of chronic conditions	Difference at time zero (t=0)	0.40 (0.28 to 0.51)	<0.001
	Pre-index linear slope difference	0.02 (0.00 to 0.03)	0.008
	Post-index linear slope difference	0.02 (0.00 to 0.03)	0.024
	Post-index quadratic slope difference	0.00 (-0.00 to 0.00)	0.629
Depressive symptom score	Difference at time zero (t=0)	0.35 (0.23 to 0.47)	<0.001
	Pre-index linear slope difference	0.03 (0.01 to 0.05)	<0.001
	Post-index linear slope difference	0.00 (-0.02 to 0.02)	0.799

Parameters represent between-group differences at time zero and differences in pre-index and post-index slopes. Time zero refers to the first fall-report wave in the fall group or the corresponding pseudo-index wave in the non-fall group.

Supplementary figures

Figure A1. ELSA trajectories of ADL limitations, chronic disease burden, and depressive symptoms around the first fall-report wave when analyses are restricted to participants with at least 3 follow-up waves.

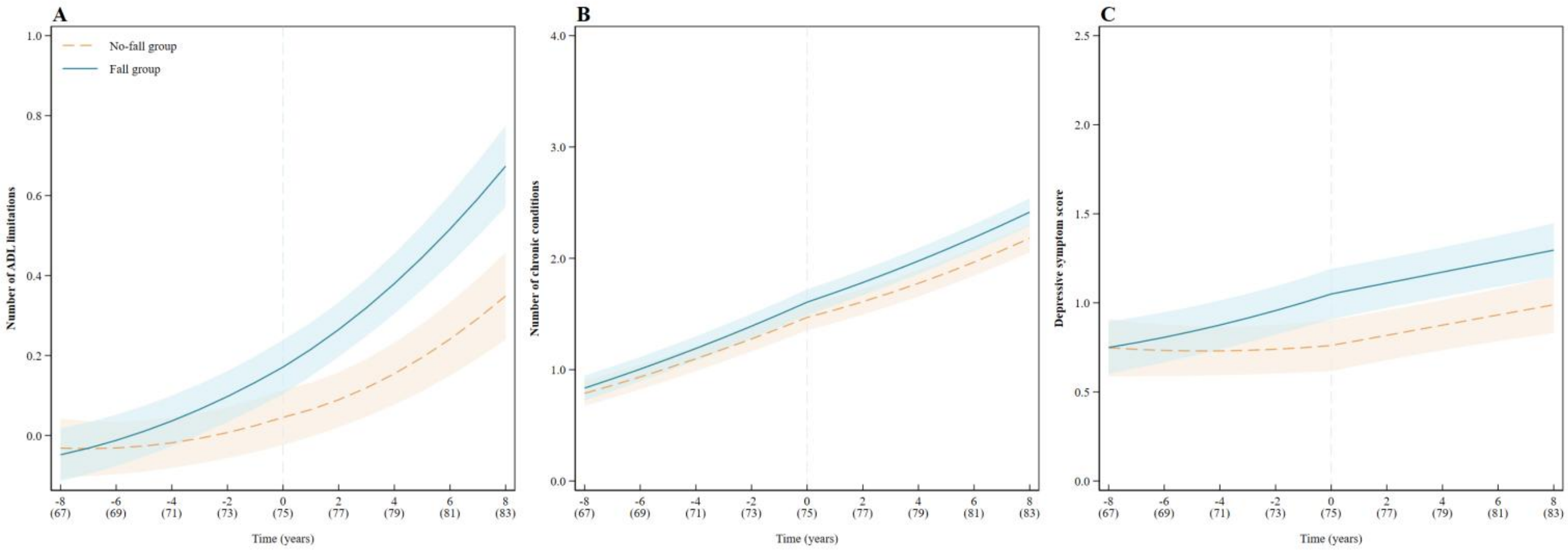

Panel A shows ADL limitations, panel B shows number of chronic conditions, and panel C shows depressive symptom score. Solid blue lines indicate the fall group and dashed orange lines indicate the non-fall group. Shaded areas indicate 95% confidence intervals.

Figure A2. ELSA trajectories of ADL limitations, chronic disease burden, and depressive symptoms around the first fall-report wave when analyses are restricted to participants with at least 4 follow-up waves.

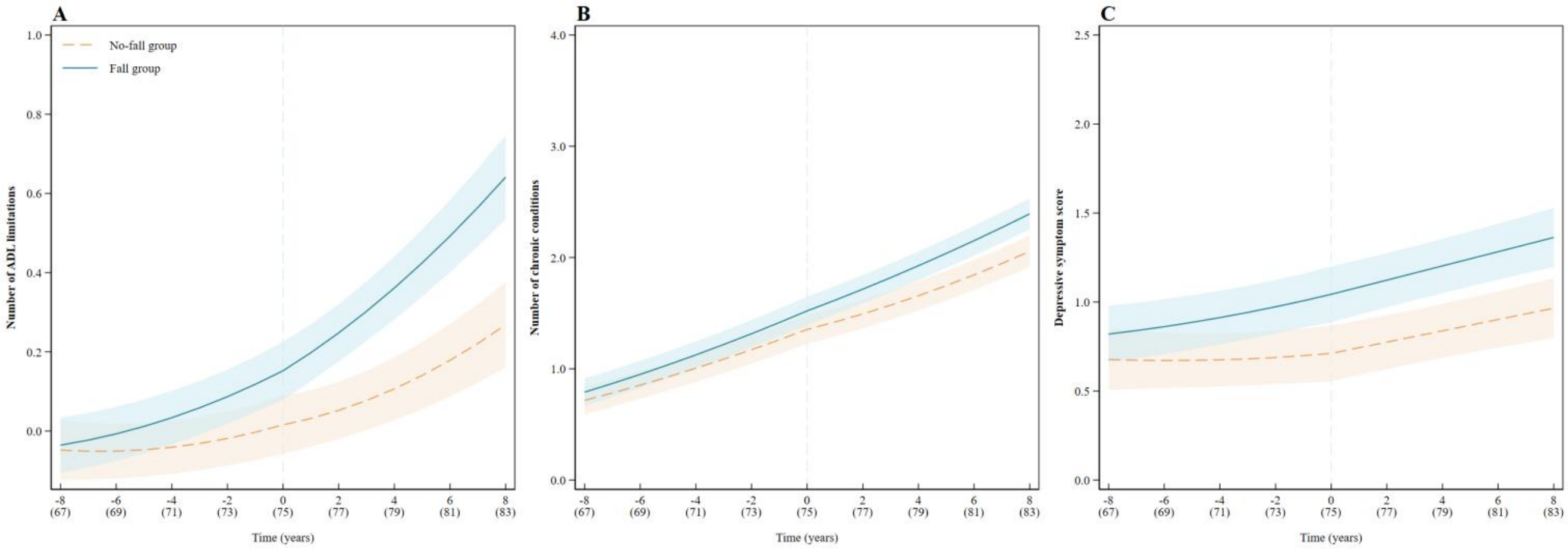

Panel A shows ADL limitations, panel B shows number of chronic conditions, and panel C shows depressive symptom score. Solid blue lines indicate the fall group and dashed orange lines indicate the non-fall group. Shaded areas indicate 95% confidence intervals.

Figure A3. ELSA trajectories of ADL limitations, chronic disease burden, and depressive symptoms around the first fall-report wave when analyses are restricted to participants with at least 5 follow-up waves.

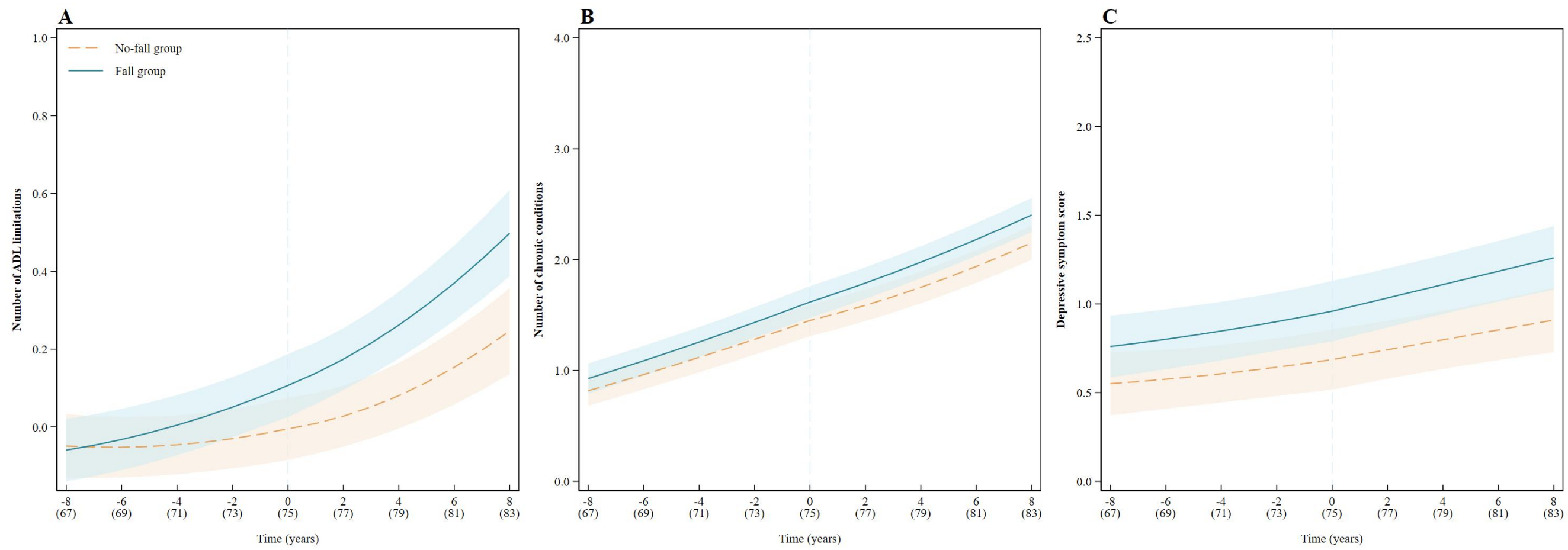

Panel A shows ADL limitations, panel B shows number of chronic conditions, and panel C shows depressive symptom score. Solid blue lines indicate the fall group and dashed orange lines indicate the non-fall group. Shaded areas indicate 95% confidence intervals.

Figure A4. HRS trajectories of ADL limitations, chronic disease burden, and depressive symptoms around the first fall-report wave when analyses are restricted to participants with at least 3 follow-up waves.

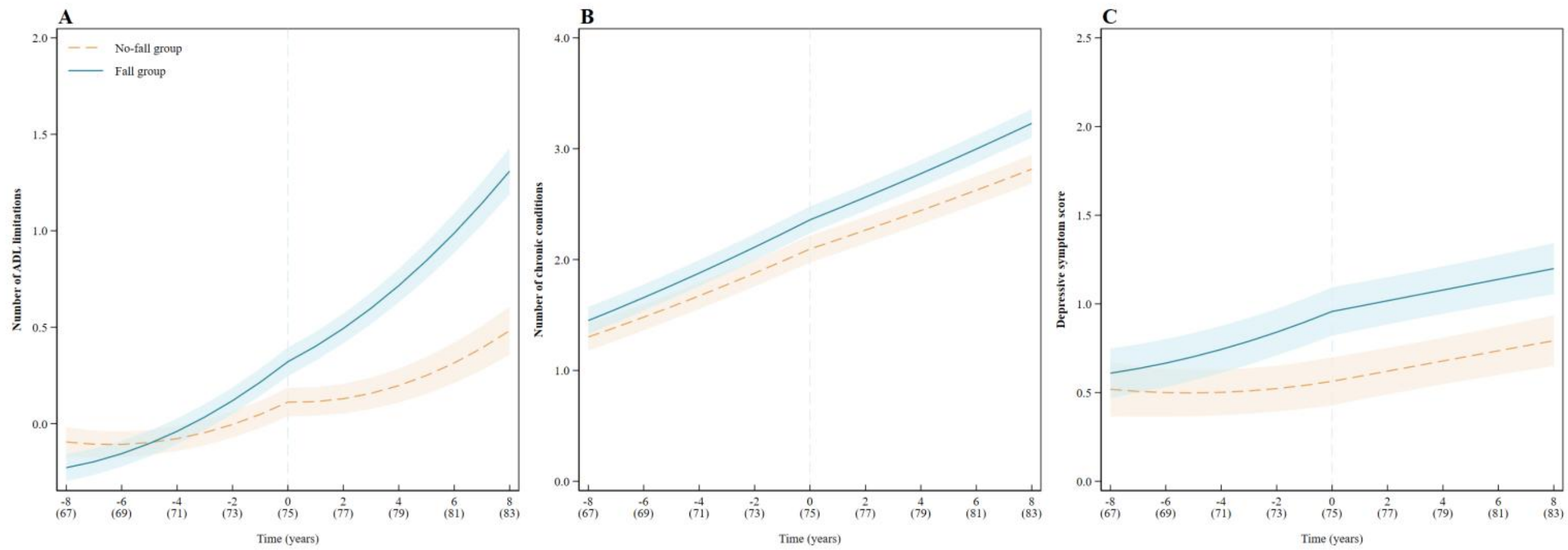

Panel A shows ADL limitations, panel B shows number of chronic conditions, and panel C shows depressive symptom score. Solid blue lines indicate the fall group and dashed orange lines indicate the non-fall group. Shaded areas indicate 95% confidence intervals.

Figure A5. HRS trajectories of ADL limitations, chronic disease burden, and depressive symptoms around the first fall-report wave when analyses are restricted to participants with at least 4 follow-up waves.

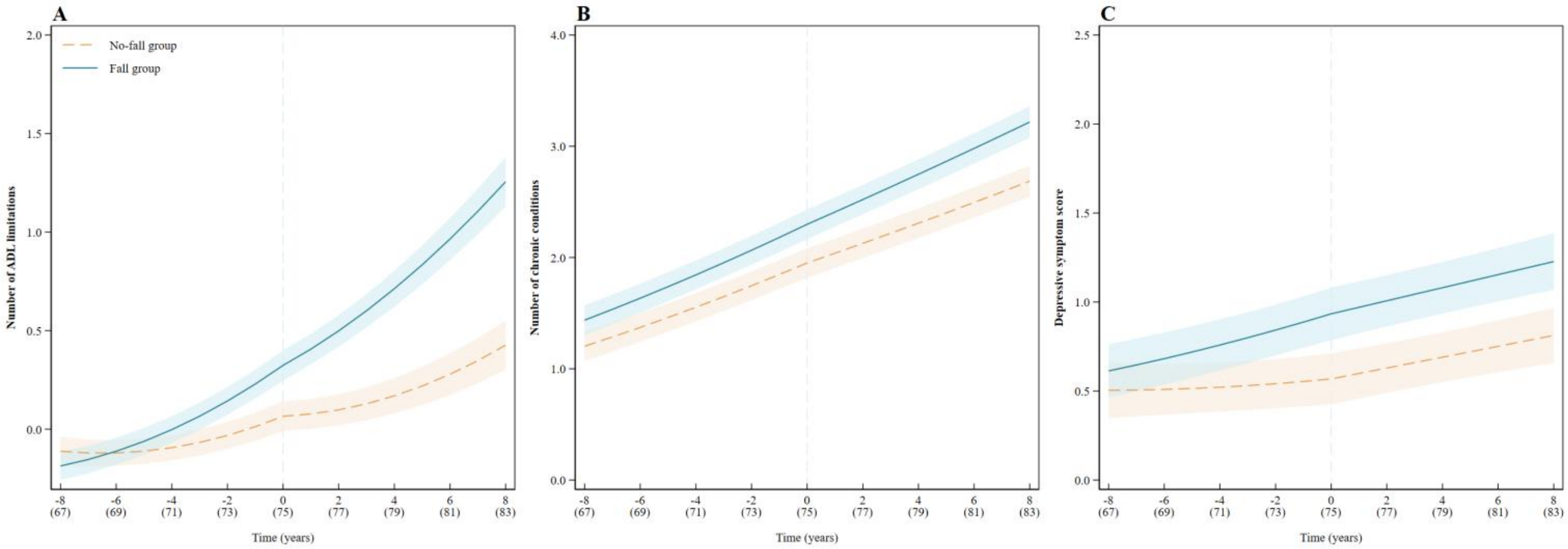

Panel A shows ADL limitations, panel B shows number of chronic conditions, and panel C shows depressive symptom score. Solid blue lines indicate the fall group and dashed orange lines indicate the non-fall group. Shaded areas indicate 95% confidence intervals.

Figure A6. HRS trajectories of ADL limitations, chronic disease burden, and depressive symptoms around the first fall-report wave when analyses are restricted to participants with at least 5 follow-up waves.

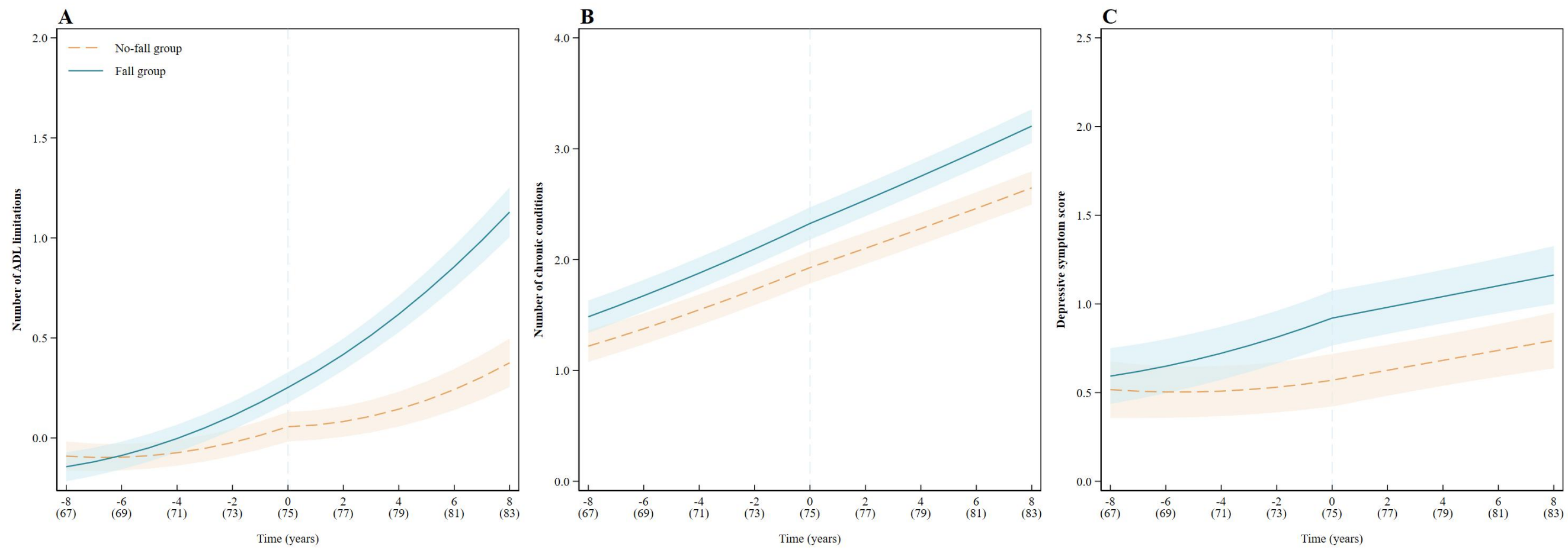

Panel A shows ADL limitations, panel B shows number of chronic conditions, and panel C shows depressive symptom score. Solid blue lines indicate the fall group and dashed orange lines indicate the non-fall group. Shaded areas indicate 95% confidence intervals.

References

1. Hedeker D, du Toit SHC, Demirtas H, Gibbons RD. A note on marginalization of regression parameters from mixed models of binary outcomes. Biometrics. 2018;74(1):354-361.
